# Supplementary figures and images for: Modeling historic incidence trends implies early field cancerization in esophageal squamous cell carcinoma
Source: PLoS Comput Biol. 2021 May 3;17(5):e1008961. doi: 10.1371/journal.pcbi.1008961 (PMC8118544; doi:10.1371/journal.pcbi.1008961)

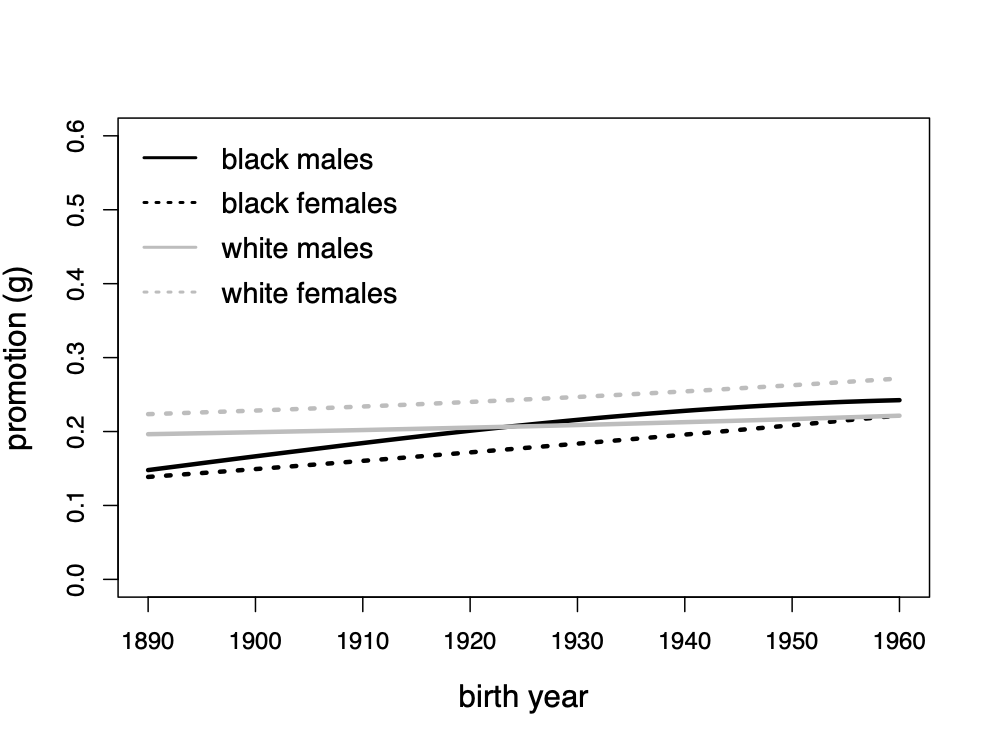

Supplement: S1 Fig — Estimated cell proliferation parameter g as a function of birth cohort. (TIF) [file pcbi.1008961.s003.tif]

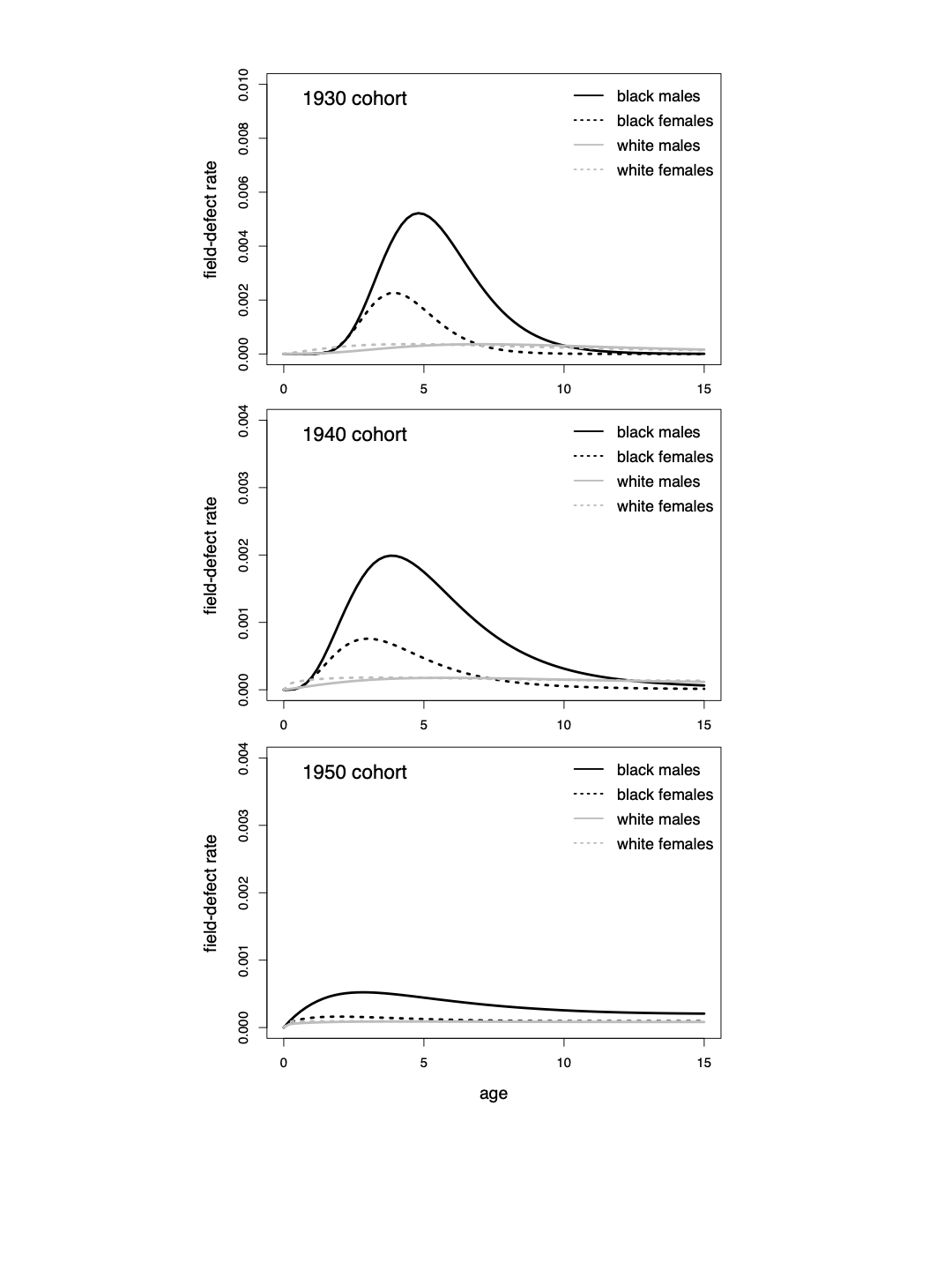

Supplement: S2 Fig — For the 1930, 1940 and 1950 birth cohort for both sexes and races. (TIF) [file pcbi.1008961.s004.tif]

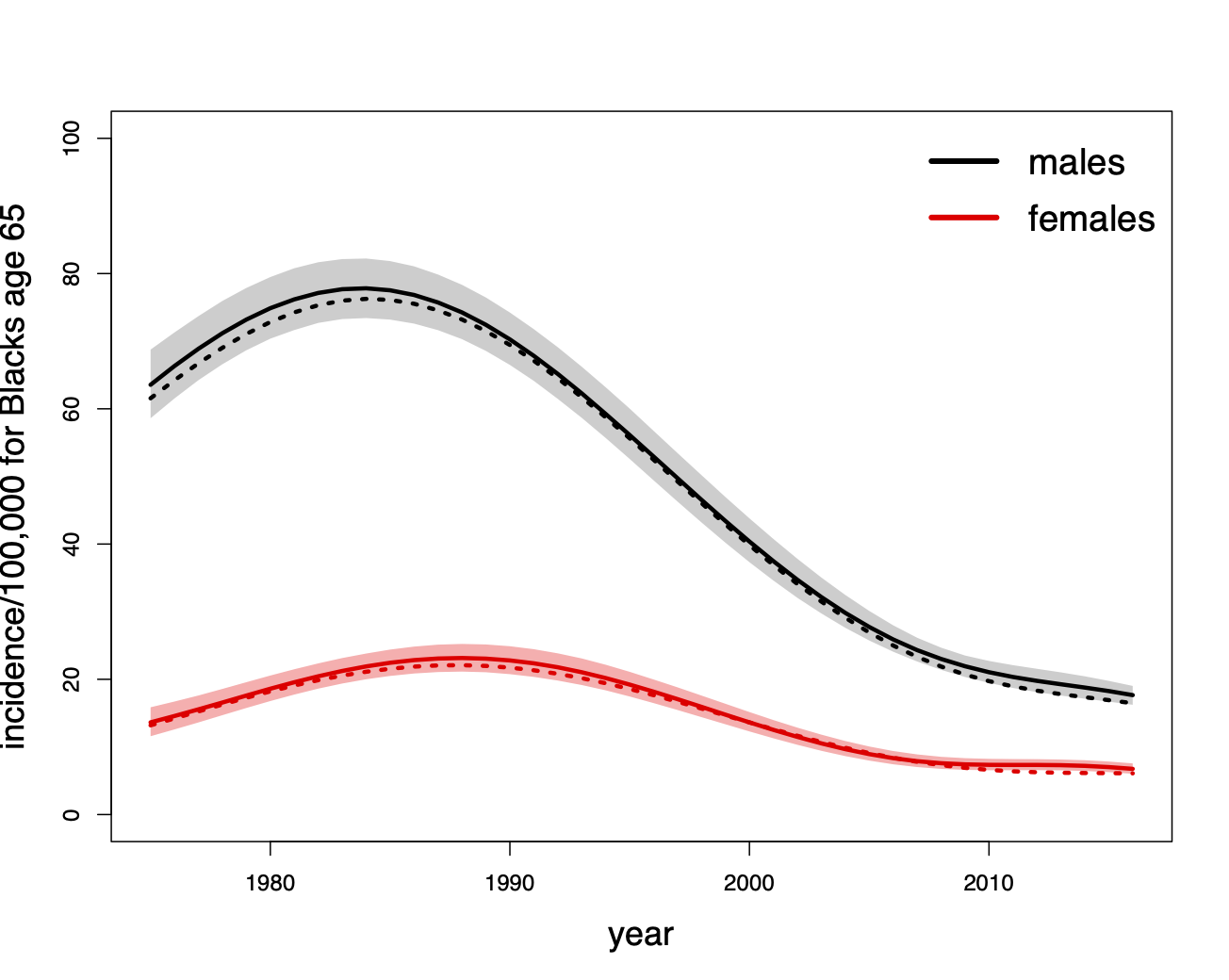

Supplement: S3 Fig — ESCC incidence between 1975 and 2016 for black males and females at age 65. Similar results for Whites (not shown). Bands indicate 95% MCMC-based credibility regions. (TIF) [file pcbi.1008961.s005.tif]
