# Supplementary material for: Modeling historic incidence trends implies early field cancerization in esophageal squamous cell carcinoma
Source: PLoS Comput Biol. 2021 May 3;17(5):e1008961. doi: 10.1371/journal.pcbi.1008961 (PMC8118544; doi:10.1371/journal.pcbi.1008961)
Supplement: Attachment — Submitted filename: Rebuttal.pdf [file pcbi.1008961.s007.pdf]

Dominik Wodarz
Associate Editor
PLOS Computational Biology

Florian Markowetz
Deputy Editor
PLOS Computational Biology

Dear Profs. Wodarz and Markowetz

We appreciate the thoughtful comments and suggestions by the reviewers, and the opportunity to revise the manuscript. Please see our responses to the reviewers' comments below. We hope the revised manuscript is acceptable for publication.

Sincerely,

Georg Luebeck and co-authors

Reviewer #1:

Based on the results of model fits to historic SEER data the authors propose an intriguing hypothesis on the appearance of field cancerization early in life (around age 10) which eventually turns into ESCC after many decades. The effect is most pronounced in black males and disappears in birth cohorts after the 1960s. An amazing finding is concerned with the narrow time window of 5-10 years in early life which may shape a field effect. Importantly, such biologically relevant trends can best be revealed by well-designed MSCE models

We agree. The 'narrow time window' for the FD, so early in life, and for all 4 cohorts analyzed, is indeed intriguing.

1) As any model the FD/MSCE model has some limitations. Although intriguingly depicted in Fig. 3, the spatial extend of the FD cannot be produced by the model which is based on exclusively age-dependent stochastic equations of motion. Moreover, information on exposure to environmental agents is not contained in SEER data so that the origin of the pronounced peaks of the FD rate in childhood can only be subject to speculation. In the model do all individuals with ESCC show a field effect or is there a distribution?

The reviewer is correct. The spatial extend and distribution of the FD cannot be inferred from cancer incidence data alone. What can be inferred is the product of the overall size of the field (in terms of the number of cells) and the first mutation rate (X' \mu_1) as indicated in Fig.3. Clearly, the predicted peaks are to be considered an inference under the model and would

need to be validated by appropriate biomarker or clinical studies, preferably in children or young adults. However, such studies would be difficult to carry out. Our study supports the idea of field-cancerization in ESCC development and suggests that one should consider (epi)genetic tissue alterations associated with nutritional deficiencies and early exposures to environmental contaminants as potential risk factors for ESCC.

The model does assume that all individuals with ESCC developed the FD, which is a simplification and a limitation of the model (now acknowledged in the discussion). However, the presence of a non-FD pathway (or pathways) to ESCC should tend to wash out the FD which we infer to be rather pronounced. Furthermore, while the non-FD (independent stem cell) model fits the data equally well, the inferred (per cell) mutation rates are implausible.

2) Peaks in the FD rate almost disappear for birth cohorts younger than 1955. Is this a real biological effect or must it be attributed to the lack of older cases in the SEER data? I suggest to add a brief summary of the SEER data with number of person years, observation period and mean age of cases.

The FD peak positions and their changing amplitudes are robust estimates. The 'disappearance' of the peak is a consequence of the very strong log-cubic (period) envelope that 'dies out' at the (estimated!) reference year, y_0 (Fig.2) and largely explains the decreasing ESCC incidences over time (Fig.4). Constant envelopes (essentially no changes in amplitude) are soundly rejected for all 4 race-gender cohorts analyzed using a likelihood ratio test. This finding is now mentioned in the Results. The attenuation of the amplitudes with more recent cohorts is unlikely an artifact due to the smaller number of cases since the MSCE model naturally yields lower incidences at younger ages and is informed by all cohorts in the data. Note, as stated, we also restricted the analysis to birth cohorts <= 1960 with several hundred ESCC cases in the more recent (1950-1960) cohorts for black and white males. A summary table (S1 Table) for the SEER9 ESCC incidence cases (1975-2016) by sex and race together with the available person years is now included in the SI.

3) The adjustment function for the FD rate involves quadratic and cubic polynomials for both calendar period and birth cohort. Experience shows that such functions are prone to weird behaviour in the fitting process notably at the limits of the data space. In contrast, the fitted FD rates appear rather regular and well behaved. Do additional regularization rules apply? A bit more explanation of the fitting procedure with respect to the FD rate would be helpful.

The reviewer makes an important point, which we failed to emphasize in Methods. To avoid potentially weird behavior of the FD rate into the future, but to capture period trends in the past (for which we have cohort-wise data), we assumed that the FD rate stays constant for years > y_0, where y_0 is the reference point beyond which the trends vanish smoothly (quadratic). This does not mean that certain low-order period trends could not be present after the last year in the SEER registry (2016 in this case), as long as the trends occur for years $y < y_0$. We now emphasize this important point. Weirdness, of course, could well arise in the past, for years prior to 1890. However, our main interest is the identification of period trends in the

time/period window for which we have observations. We clarified this important point in Methods.

4) Are the projected peaks in the FD rate still clinically relevant given their disappearance in persons born after 1960 and decreasing trends of ESCC? Should they be examined in children today or are they only of historical interest?

We appreciate this question which addresses the potential relevance of our prediction for controlling ESCC, in particular for US Blacks who continue to experience a higher ESCC incidence (about 2x) than US Whites. Although the early-in-life peaks of the FD rate are found to be decreasing with time, the FD rate for the more recent cohorts (1950+) is not zero, but is predicted by the model to be more constant. Whether this means that other exposures later in life (such as smoking and/or alcohol) come to play for these cohorts is unclear and cannot be learned from these data. Clearly, the clinical relevance of this analysis is limited, but the method may yield stronger predictions when it comes to cancers with well recognized FDs, including lung cancer, colorectal cancer, and esophageal adenocarcinoma where the FD is known as Barrett's esophagus.

Minor comments:

1) p 4, line 87: The traditional proportional hazard approach can be extended with limited effect to take into account past environmental exposure for the explanation of subsequent health risks. For example, risk estimates from distributed nonlinear models (DNLMs) have been compared with those from MSCE models for the case of lung cancer incidence from radon exposure in uranium miners. The main difference between DNLMs and MSCE models is given by the process-oriented approach implemented in stochastic differential equations for MSCE models. On the other hand, DNLMs apply a simple mdulated summation of previous exposure contributions.

We thank the reviewer for pointing out the DNLM method. A reference to this work has been included. Explicit exposure information clearly strengthens the case for models, such as MSCE or DLNM, that allow the direct incorporation of exposures (rates or cumulative) into their framework. Unfortunately, registry data in general lack this information so indivdual environmental exposures and relevant life-style factors are unknown. However, when this information is known or can be inferred for specific subpopulations (e.g. historic smoking uptake and prevalence) , it can be directly incorporated into our model and the incidence trends linked to responsive biological processes in cancer.

2) In Table 1 the median of parameter estimates are given. Do the figures also show the medians? How large is the model uncertainty? Can it be displayed in the figures with 95% CIs or is it too small to show?

The fits shown (Fig.4-6) are based on maximum likelihood estimates which are very close to the (marginal) MCMC medians. The rationale to report MCMC-based medians together with their

95% credibility regions was to provide a better sense of the stability of the parameter estimates. To demonstrate how this (posterior) uncertainty plays out for the estimated ESCC incidence trends across the years of ESCC diagnosis (1975-2016), we now provide an example in S3 Fig: ESCC incidence between 1975 and 2016 for black males and females at age 65. This graph shows very similar incidence estimates for MLE-based and MCMC-based (median) predictions of ESCC for Blacks with tight point-wise 95% credibility regions. We find similar results for Whites (not shown).

3) p9, line 208: Martincorena et al. Science 2018 report non-localized clones of cells harboring NOTCH1 and TP53 mutations all over the epithelium of the esophagus. Since these oncogenes are also found in full blown cancers their findings would not support the existence of well-delineated premalignant fields as early lesions. This fact would not speak against the validity of the present MSCE model which actually does not rely on localized field defects as suggested in Fig 3. On the other hand, field defect prevalence levels from age 10 (Fig. 6) whereas the mutation burden per cell increases linearly with age (Fig. 1C of Martincorena et al. 2018). Is it possible to interpret the results of Martincorena et al. 2018 in the framework of the MSCE model?

We don't know the answer to this question but would point out that not all mutations seen in normal esophageal tissues are showing up with similar frequency in the cancers. TP53 and NOTCH1 mutations are a case in point since the vast majority of ESCC do harbor mutations in TP53 but not NOTCH1 even though NOTCH1 mutations are rather prevalent in normal esophageal tissue. It is possible that the FD represent a punctuated selection of TP53 defects, maybe early in life as our model suggests. In fact, TP53 mutations have been implicated by others to be contributing to field cancerization in esophagus (see reference Tian, D., Feng, Z., Hanley, N. M., Setzer, R. W., Mumford, J. L., & DeMarini, D. M. (1998). Multifocal accumulation of p53 protein in esophageal carcinoma: Evidence for field cancerization. International Journal of Cancer, 78(5), 568–575.)

4) p 11, line 230: Goodness-of-fit seems to have been measured by deviance which has been compared with the deviance from a model with multiplicative adjustment of secular trends. Could you provide a table of deviance and AIC for both model versions in the Supplement. It would help assess the new modeling approach. Is there much overdispersion in the data which would be revealed by the Poisson deviance?

We appreciate the suggestion and have included a Table in the SI (S2 Table) that provides the Poisson deviances of the models that we fitted to each of 2043 age-period strata for the 4 populations studied. This Table shows that, with the exception of males, the regularized FD model is preferable to the saturated Poisson model. The saturated model has p-values of 0.034 and 0.07 for black males and white males, respectively. Thus, the FD model cannot be soundly rejected on statistical grounds, even for males! The table also confirms that there is generally little difference in the deviances between non-FD (stem cell independence) and FD models. Regarding overdispersion, the rule of thumb is that the ratio of residual deviance to df is larger 1. Based on this rule only males show mild overdispersion (1-dev/df < 6%).

5) It would improve the readability and facilitate reproducibility if units were added to the model parameters in the figures and in Table 1.

Done

Misprints:
p2, line 19: missing reference

fixed

p 3, line 72: replace hazard rate by FD rate

fixed

p 4, line 106: is B_0 fixed to 1800?

We thank the reviewer for pointing out this omission. We chose to set $B_0=y_0$ as we did not find fits with significant likelihood improvements with an independent parameterization for B_0 and y_0.

p 5, top: brackets are missing in g = ….

fixed

p5, Fig 2: do the coefficients a, b of the polynomials correspond to w_1, w_2 in the parameter Table 1?

Thank you for pointing this out. We have revised Fig.2 and replaced *a* and *b* with the parameters w_1 and w_2 , as defined in the figure.

p 12, line 256, remove Parameter

fixed

Reviewer #2:
The conclusions from this analysis offer interesting aspects of the etiology of this disease that are useful to pursue for a better understanding of the etiology. Having said that, an important limitation of this work is that there are still aspects of the problem that are not resolved. While various versions of the multistage carcinogenesis model seem to work well for many cancer sites, it is difficult to verify all of the details at the cellular level. In addition, the data are not available for identifying the specific factors that are at play in giving rise to these different trends. For

example, the results suggest that the field-defect rate has changed for cohorts in ways the differ by race and sex (Figure 5) but at this time we do not have the data that would point to the reasons for these changes.

The reviewer's point is well taken. Although the model is consistent with the observed ESCC incidence trends in US SEER, the nature and causes of the assumed field-defect and its age-period and cohort dependences remain hypothetical. What is surprising, however, is that the predicted FD did not show an age or period signature that reflects smoking initiation and/or smoking prevalence by race. The effects of smoking and alcohol consumption on ESCC have been studied extensively, mainly among adults via association, however predisposing factors and exposures early in life have not. A strength of this modeling approach is that it distinguishes early (initiating) from late (tumor promoting) events and therefore provides an analytical tool to study the consequences of early vs late effects on cancer risk. It is a surprise, unlike other applications of the MSCE model to lung cancer - that the predicted ESCC incidence trends did not reflect historic smoking (e.g. smoking initiation) and alcohol-use related trends in the US, in particular when it comes to racial differences that are strongly period-dependent. Although this could be a failure of the model, there is evidence that early-in-life exposures and nutritional deficiencies (which may well be linked to smoking and alcohol consumption later in life), are important risk factors for ESCC linked to poverty and other socioeconomic disparities in the US.
~~In short, we believe the value of the approach introduced here is that it generating an interesting hypothesis that hopefully will motivate epidemiologists and clinicians to identify biomarkers for field-defects that may be arise early in life.~~

A puzzling aspect of this manuscript is the claim that this method disentangles the identifiability problem in age-period-cohort models (p2. l.20). This is a statistical model which is linear with additive contributions for each of the time elements. A log transformation is commonly used for disease rates, so this is equivalent to a multiplicative model, as stated by the authors. Period is the sum of age and cohort, so in a way the model considered in this manuscript includes all these temporal elements. However, in the statistical model one is trying to estimate effects identified with each of the temporal elements. In this development, the progression of disease is considered for each cohort, so that the underlying effects are associated with age and cohort. It not clear what contribution is uniquely attributable to period. Such an effect might affect all cohorts at a particular time, but there is nothing like this in the model, or at least this is not obvious. Period appears to be identified with "regularization", but it would be helpful to explain more fully what this is and how it comes into the overall model. Figure 2 needs more detail, such as a scale for the vertical axis. What do the lines in the graph represent? Are these for different values of a and b? Do we have such a graph for each B, for example?

We apologize for the lack of clarity. The biological convolution model presented (convolving a regularized field-defect with a multistage clonal expansion model for subsequent processes that occur within an established FD) differs from the statistical APC model in that the age-effect is constrained by the biological processes which are non-linear (specifically we model this effect as

a multi-type branching process). This finesses the fundamental identifiability problem in statistical APC models, which we did not use for this analysis. Furthermore, the term ‘period’ in this analysis does not refer to year-of-diagnosis but refers to historical time which we allow to affect the FD rate irrespective of cohort. ‘Anchoring’ is achieved by removing the trends smoothly (log-quadratic-cubic) at a reference year y_0, irrespective of cohort. We have modified the Methods to clarify these issues and to avoid confusion with the unconstrained statistical models that suffer from non-identifiability.

We revised Fig.2 and its legend to clarify how the regularization of the FD rate works. We labelled the y-axis, indicated the position of the background rate, and provided illustrative trajectories for the FD rate (for a given birth cohort), one set with the logarithmic attenuating term log(s/s0) included (solid lines), and one set without it (dotted lines). These lines were randomly generated using random uniform deviates for w_1 [-1,.4] and w_2 [-1,1]. Models that included the attenuating term yielded significantly better fits by deviance.

On the one hand, the relationship with APC seems irrelevant because this a biological model that provides insight into disease trends. However, it could be an interesting aside if it does fit directly into the framework of an APC model. If it does not, I do not think that this would detract from the value of this contribution.

We agree. A comparison of the biological model with a (fully agnostic) statistical APC model may be of interest. However, we don’t see how it would reveal the separate dynamics of the postulated field defect, or an early initiating mutation, for that matter. Please see our response to the 4th point raised by Reviewer 1. For comparison, we’ve included deviances for (smoothed) hybrid APC model versions with cohort and period trends operating multiplicatively on the ESCC hazard function.
