# Supplementary material for: Modeling historic incidence trends implies early field cancerization in esophageal squamous cell carcinoma
Source: PLoS Comput Biol. 2021 May 3;17(5):e1008961. doi: 10.1371/journal.pcbi.1008961 (PMC8118544; doi:10.1371/journal.pcbi.1008961)
Supplement: Attachment — Submitted filename: PCOMPBIOL-D-20-02290-review.docx [file pcbi.1008961.s006.docx]

Review of PCOMPBIOL-D-20-02290 «Modeling historic incidence trends implies early field cancerization in esophageal squamous cell carcinoma» by Luebeck et al.

In the U.S. the cancer incidence for the esophagus with squamous cell histology (ESCC) decreases in contrast to increasing incidence of adenocarcinoma (EAC). Earlier work of Jeon et al. 2006 investigated the influence of field cancerization (termed Barrett›s esophagus) on EAC incidence rates for U.S. whites with tabulated data from the SEER registry. A cell-based Multi-Stage Clonal Expansion (MSCE) model was combined with a density function for a premalignant field defect (FD) depending solely on age. Compared to «standard» models MSCE models with a FD component produce biologically plausible values for FD rates in the range of 10^-5^-10^-4^ per year whereas mutation rates in standard MSCE models come out too low by about five orders of magnitude. In Jeon et al. 2006 adjustment for secular trends of calendar year and birth cohort were performed by multiplicative factors to the «pure» hazard function from the MSCE model which depended only on age. Both versions of MSCE models describe the data almost equally well.

The present work presents a new approach of secular adjustment directly in biological model parameters, notably in the FD rate and in the rate of clonal expansion. Fig. 2 depicts an adjustment function depending on calendar period and attained age which is supplemented with a birth year dependence.

Based on the results of model fits to historic SEER data the authors propose an intruiging hypothesis on the appearance of field cancerization early in life (around age 10) which eventually turns into ESCC after many decades. The effect is most pronounced in black males and disappears in birth cohorts after the 1960s. An amazing finding is concerned with the narrow time window of 5-10 years in early life which may shape a field effect. Importantly, such biologically relevant trends can best be revealed by well-designed MSCE models

Comments:

1) As any model the FD/MSCE model has some limitations. Although intruigingly depicted in Fig. 3, the spatial extend of the FD cannot be produced by the model which is based on exclusively age-dependent stochastic equations of motion. Moreover, information on exposure to environmental agents is not contained in SEER data so that the origin of the pronounced peaks of the FD rate in childhood can only be subject to speculation. In the model do all individuals with ESCC show a field effect or is there a distribution?

2) Peaks in the FD rate almost disappear for birth cohorts younger than 1955. Is this a real biological effect or must it be attributed to the lack of older cases in the SEER data? I suggest to add a brief summary of the SEER data with number of person years, observation period and mean age of cases.

3) The adjustment function for the FD rate involves quadratic and cubic polynomials for both calendar period and birth cohort. Experience shows that such functions are prone to weird behaviour in the fitting process notably at the limits of the data space. In contrast, the fitted FD rates appear rather regular and well behaved. Do additional regularization rules apply? A bit more explanation of the fitting procedure with respect to the FD rate would be helpful.

4) Are the projected peaks in the FD rate still clinically relevant given their disappearance in persons born after 1960 and decreasing trends of ESCC? Should they be examined in children today or are they only of historical interest?

Minor comments:

1) p 4, line 87: The traditional proportional hazard approach can be extended with limited effect to take into account past environmental exposure for the explanation of subsequent health risks. For example, risk estimates from distributed nonlinear models (DNLMs) have been compared with those from MSCE models for the case of lung cancer incidence from radon exposure in uranium miners. The main difference between DNLMs and MSCE models is given by the process-oriented approach implemented in stochastic differential equations for MSCE models. On the other hand, DNLMs apply a simple mdulated summation of previous exposure contributions.

2) In Table 1 the median of parameter estimates are given. Do the figures also show the medians? How large is the model uncertainty? Can it be displayed in the figures with 95% CIs or is it too small to show?

3) p9, line 208: Martincorena et al. Science 2018 report non-localized clones of cells harboring NOTCH1 and TP53 mutations all over the epithelium of the esophagus. Since these oncogenes are also found in full blown cancers their findings would not support the existence of well-delineated premalignant fields as early lesions. This fact would not speak against the validity of the present MSCE model which actually does not rely on localized field defects as suggested in Fig 3. On the other hand, field defect prevalence levels from age 10 (Fig. 6) whereas the mutation burden per cell increases linearly with age (Fig. 1C of Martincorena et al. 2018). Is it possible to intterpret the results of Martincorena et al. 2018 in the framework of the MSCE model?

4) p 11, line 230: Goodness-of-fit seems to have been measured by deviance which has been compared with the deviance from a model with multiplicative adjustment of secular trends. Could you provide a table of deviance and AIC for both model versions in the Supplement. It would help assess the new modeling approach. Is there much overdispersion in the data which would be revealed by the Poisson deviance?

5) It would improve the readability and facilitate reproducibility if units were added to the model parameters in the figures and in Table 1.

Misprints:

p2, line 19: missing reference

p 3, line 72: replace hazard rate by FD rate

p 4, line 106: is B_0_ fixed to 1800?

p 5, top: brackets are missing in g = ….

p5, Fig 2: do the coefficents a, b of the polynomials correspond to w_1,_ w_2_ in the parameter Table 1?

p 12, line 256, remove Parameter

Reference

Assenmacher et al., Exposure–lag–response associations between lung cancer mortality and radon exposure in German uranium miners, Radiation and Environmental Biophysics (2019) 58:321–336, https://doi.org/10.1007/s00411-019-00800-6
